# The Expression of Pyroptosis-Related Gene May Influence the Occurrence, Development, and Prognosis of Uterine Corpus Endometrial Carcinoma

**DOI:** 10.3389/fonc.2022.885114

**Published:** 2022-04-29

**Authors:** Xiaoling Huang, Yangyi Li, Jiena Li, Xinbin Yang, Jianfeng Xiao, Feng Xu

**Affiliations:** ^1^ Department of Respiratory and Critical Care Medicine, The First Affiliated Hospital of Shantou University Medical College, Shantou, China; ^2^ Department of Obstetrics and Gynecology, Heze Municipal Hospital, Heze, China; ^3^ Department of Thoracic Surgical Oncology, The Affiliated Cancer Hospital and Institute of Guangzhou Medical University, Guangzhou, China; ^4^ Department of Reproductive Center, The First Affiliated Hospital of Shantou University Medical College, Shantou, China

**Keywords:** pyroptosis, immune checkpoint inhibitor, uterine corpus endometrial carcinoma, prognostic risk model, tumor occurrence and development

## Abstract

**Background:**

Increasing evidence has demonstrated that pyroptosis exerts key roles in the occurrence, development, and prognosis of uterine corpus endometrial carcinoma (UCEC). However, the mechanism of pyroptosis and its predictive value for prognosis remain largely unknown.

**Methods:**

UCEC data were acquired from The Cancer Genome Atlas (TCGA) database. The differentially expressed genes in UCEC vs. normal cases were selected to perform a weighted correlation network analysis (WGCNA). Forty-two UCEC-associated pyroptosis-related genes were identified *via* applying differential expression analysis. Protein–protein interaction (PPI) and gene correlation analyses were applied to explore the relationship between 21 UCEC key genes and 42 UCEC-associated pyroptosis-related genes. The expression of 42 UCEC-associated pyroptosis-related genes of different grades was also calculated. The immune environment of UCEC was evaluated. Furthermore, pyroptosis-related genes were filtered out by the co-expression. Univariate and a least absolute shrinkage and selection operator (LASSO) Cox analyses were implemented to yield a pyroptosis-related gene model. We also performed consensus classification to regroup UCEC samples into two clusters. A clinically relevant heatmap and survival analysis curve were implemented to explore the clinicopathological features and relationship between two clusters. Furthermore, a Kaplan–Meier survival analysis was implemented to analyze the risk model.

**Results:**

Twenty-one UCEC key genes and 42 UCEC-associated pyroptosis-related genes were identified. The PPI and gene correlation analysis showed a clear relationship. The expression of 42 UCEC-associated pyroptosis-related genes of different grades was also depicted. A risk model based on pyroptosis-related genes was then developed to forecast overall survival among UCEC patients. Finally, Cox regression analysis verified this model as an independent risk factor for UCEC patients.

**Conclusions:**

The expression of pyroptosis-related gene may influence UCEC occurrence, development, and prognosis.

## Introduction

Uterine corpus endometrial carcinoma (UCEC), a gynecologic cancer with the second highest incidence rate in women, is the main cause of death in female patients with cancers because of the high relapse rate (1, 2). UCEC has a low survival rate and poor prognosis (3–5). However, if diagnosed correctly in the early stage, 5-year survival rates up to 90% have been reported (6). A growing number of studies have shown that some patients in the same stage can exhibit different clinical outcomes and characteristics; therefore, an urgent need for increasingly valid and accurate methods to diagnose and treat patients with UCEC exists (7).

Pyroptosis is a newly discovered and confirmed mode of programmed cell death (8–10). Pyroptosis can be divided into classical and non-classical pathways. Pyroptosis relies mainly on inflammatory vesicles activating some proteins of the caspase family, namely, the pore-forming proteins gasdermin D (*GSDMD*) or gasdermin E (*GSDME/DFNA5*), causing cleavage, activation, and translocation of activated gasdermin proteins to the membrane, a site at which they form pores, cause cell swelling and cytoplasmic efflux, and eventually lead to cell membrane rupture (11). Pyroptosis is thought to contribute a dual role in tumorigenesis and progression *via* restraining tumorigenesis and progression and creating a microenvironment that feeds the cancer and accelerates its growth (12). As found in non-small cell lung cancer, the transcription factor, p53, impedes tumor progression by promoting pyroptosis (13). In previous studies addressing gastric cancer, a novel gene signature associated with pyroptosis has been identified as a predictor of prognosis (14). Chemotherapy-induced *GSDME*-mediated pyroptosis plays a positive prognostic role in the antitumor response in oral squamous cell carcinoma (15). However, the value of genes associated with pyroptosis in UCEC patients has not yet been explored.

In our study, pyroptosis-related genes were filtered out to explore their correlation with the occurrence and development of UCEC. A risk model was constructed *via* application of the bioinformatic and statistical analyses of data from UCEC patients. Moreover, we estimated its predictive value among patients with UCEC.

## Methods

### Data Source

The clinical information and transcriptome profiles of UCEC were acquired separately from The Cancer Genome Atlas (TCGA) database. To reduce statistical bias in our analysis, UCEC patients with missing overall survival (OS) values were excluded. As a result, we acquired a TCGA data set comprising 549 patients.

### Selection of Pyroptosis-Related Genes in Uterine Corpus Endometrial Carcinoma

The expression matrices of pyroptosis-related genes were retrieved according to previous studies. A Wilcoxon test was used to analyze the differential expression of 47 pyroptosis-related genes in UCEC patients and control patients. The translational-level validation of pyroptosis-related genes was carried out by The Human Protein Atlas database (https://www.proteinatlas.org/).

### Identification of Uterine Corpus Endometrial Carcinoma Key Genes

Differentially expressed genes (DEGs) were detected in UCEC vs. controls using the R package “limma.” After correction by the false discovery rate (FDR), the P value <0.01 and |log2 fold change (FC)| >2 were applied for DEG screening. DEGs were used to identify key UCEC genes, and this process was performed using a weighted correlation network analysis (WGCNA) for mRNA expression data. While the power was equal to 6 (*R* ^2^= 0.9), eight modules were obtained. According to the cor. Tumor > 0.2 and the cor. module membership > 0.8, 21 UCEC key genes were acquired.

### Visualization of Immune Cells in Tissue Infiltration

R package “CIBERSORT.R” was used to evaluate the proportion of 22 types of immune cells that was used as a reference expression signature with 1,000 permutations. The R packages, “corrplot,” “vioplot,” “ggplot2,” and”dplyr” were used to complete this process.

### Construction of the Risk Model

Least absolute shrinkage and selection operator (LASSO) Cox regression was performed using “glmnet” in the R package. Application of the subsequent formula was used to count the risk score: Risk score = coef (gene1) × expr (gene1) + coef (gene2) × expr (gene2) + …… + coef (gene n) × expr (gene n) (16) in which coefi corresponded to the coefficients, coef (gene n) corresponds to the coefficient of pyroptosis-related genes correlated with survival, and expr (gene n) corresponded to the expression of pyroptosis-related genes. Finally, after adoption of a median risk score, high and low risk subgroups were created.

### Consensus Cluster Analysis

R package “Consensus ClusterPlus” was utilized to implement the consensus classification of UCEC, which supplies quantitative and visual consequence to calculate the number of unsupervised clusters. Sampling of 80% of the tumors 100 times was done, and k-means algorithm based on Euclidean metric was used for each cluster (17).

### Kaplan–Meier Survival and Receiver Operating Characteristic Analyses

The procedure used R packages “survminer” and “survival”, a Kaplan–Meier survival analysis to appraise OS differences between high- and low-risk groups for pyroptosis-related subtypes.

The area under the curve (AUC) per set was computed and was represented as a curve. When the curve achieved the highest point, it was defined as maximum AUC value, and the calculation process was terminated. Moreover, this model was recognized as the best option (18).

### Verification of the Independence of Risk Model

Using R package “survival” and “survminer”, multivariate and univariate Cox regression analyses, respectively, were executed to validate the function of this prognostic model when considering regular clinical features (age and tumor grade) in UCEC patients.

### Protein–Protein Interaction Analysis and Gene Ontology Enrichment Analyses

We applied the protein–protein interaction (PPI) analysis of the STRING database. R packages “colorspace”, “stringi”, and “ggplot2” completed Gene Ontology (GO) functional analysis was performed.

## Results

### Pyroptosis-Related Genes of Uterine Corpus Endometrial Carcinoma

**Figure 1:**
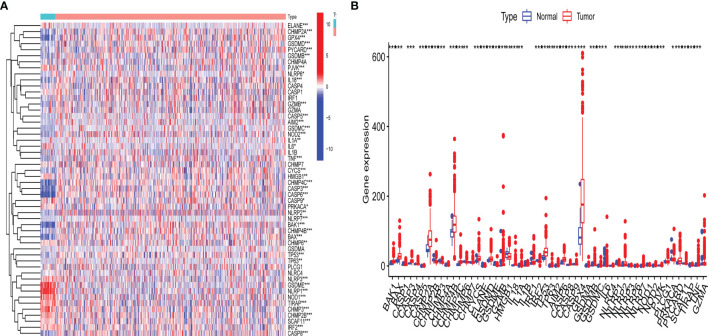
Expression of pyroptosis-related gene in uterine corpus endometrial carcinoma (UCEC). **(A)** Heatmap of expression levels of 47 pyroptosis-related genes. P-values are shown as: *p < 0.05; **p < 0.01; ***p < 0.001; **(B)** Boxplot of expression levels of 47 pyroptosis-related genes. P-values are shown as: *p < 0.05; **p < 0.01; ***p < 0.001.

The detailed workflow for subsequent analyses was shown in **Supplementary Figure S1**. A differential expression analysis for 47 pyroptosis-related genes with UCEC patients and controls was performed (**Figure 1**). Results revealed that the expression of pyroptosis-related gene may influence the occurrence of UCEC. Then, we also named these 42 pyroptosis-related genes as UCEC-associated pyroptosis-related genes.

### Identification of Differentially Expressed Genes in Uterine Corpus Endometrial Carcinoma

According to the |log2FC| > 2 and FDR < 0.01, 5,135 DEGs with 2,707 upregulated and 2,428 downregulated genes were acquired from TCGA data set.

### Identification of Key Genes

**Figure 2:**
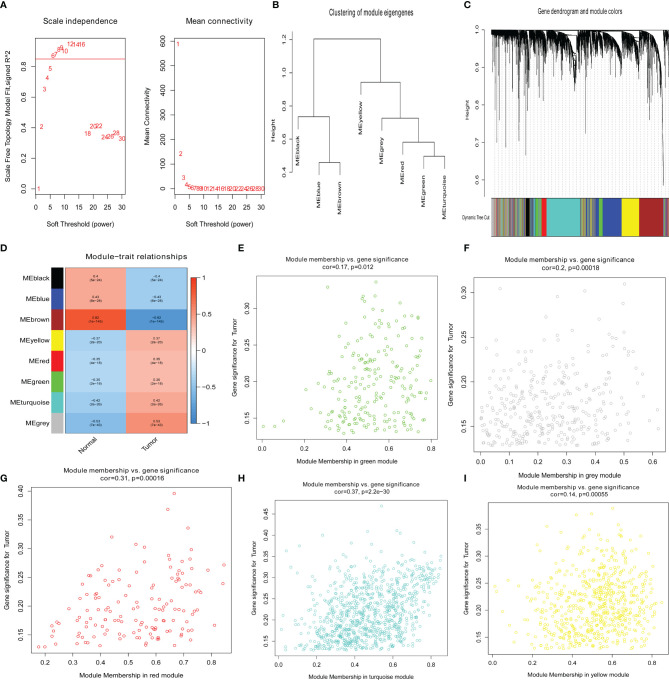
Identification of key genes *via* the weighted correlation network analysis (WGCNA). **(A)** Analysis of the scale-free fit index (left) and the mean connectivity (right) for various soft-thresholding powers. **(B)** Clustering dendrograms of genes based on a dissimilarity measure (1-TOM). **(C)** Module–trait associations were evaluated by correlations between module eigengenes and sample traits. **(D–I)** Scatterplot of gene significance for uterine corpus endometrial carcinoma (UCEC) in green, gray, red, turquoise, and yellow modules.

To confirm the key modules and genes of UCEC, we performed WGCNA for 5,135 DEGs. Based on the lowest soft threshold power 6 (**Figure 2A**) and the scale-free topology fit index 0.90 (**Figure 2A**), a hierarchical clustering tree was constructed.

To discern modules correlated with UCEC, we calculated the relationship between UCEC and each module. Subsequently, eight modules were identified (**Figure 2B**). A hierarchical clustering tree (**Figure 2C**) was constructed. It was then found that the modules significantly correlated with UCEC, which suggested that genes in these modules are mainly correlated with UCEC. According to the cor. UCEC > 0.2 and cor. module membership > 0.8, 21 genes with high connectivity in yellow, red, green, turquoise, and gray modules were screened as key UCEC genes (**Figures 2E–I**, respectively). The information concerning the 21 UCEC (**Figure 2D**) key genes was shown in **Table 1**.

**Table 1:** The information of 21 UCEC key genes.

Key Gene	Module Color	Cor.MM	Cor.Tumor	Up or Down
UQCRQ	Yellow	0.80439817	0.281223808	Down
BRMS1	Yellow	0.800267174	0.239376017	Down
AURKAIP1	Yellow	0.84914721	0.214056496	Down
R0M01	Yellow	0.820100469	0.212838856	Down
NAA38	Yellow	0.812135916	0.211460593	Down
B4GALT3	Red	0.845193224	0.27167098	Down
Clorf43	Red	0.841266343	0.239137924	Down
CCNB1	Turquoise	0.851869441	0.350618097	Down
NCAPH	Turquoise	0.848330814	0.340764398	Down
CCNB2	Turquoise	0.803022519	0.33534699	Down
KIF4A	Turquoise	0.846952695	0.321679867	Down
GINS1	Turquoise	0.819930489	0.320505357	Down
RAD51	Turquoise	0.803710105	0.318186649	Down
CDCA8	Turquoise	0.80837858	0.316062313	Down
MELK	Turquoise	0.803693514	0.307986265	Down
KIF2C	Turquoise	0.840165224	0.307280656	Down
OIP5	Turquoise	0.816450347	0.300223643	Down
RACGAP1	Turquoise	0.836889316	0.300193525	Down
NCAPG	Turquoise	0.807017039	0.295910232	Down
DLGAP5	Turquoise	0.825883301	0.290340659	Down
CCNA2	Turquoise	0.813546282	0.285855296	Down

### Exploration of the Relationship Between Pyroptosis and Uterine Corpus Endometrial Carcinoma

**Figure 3:**
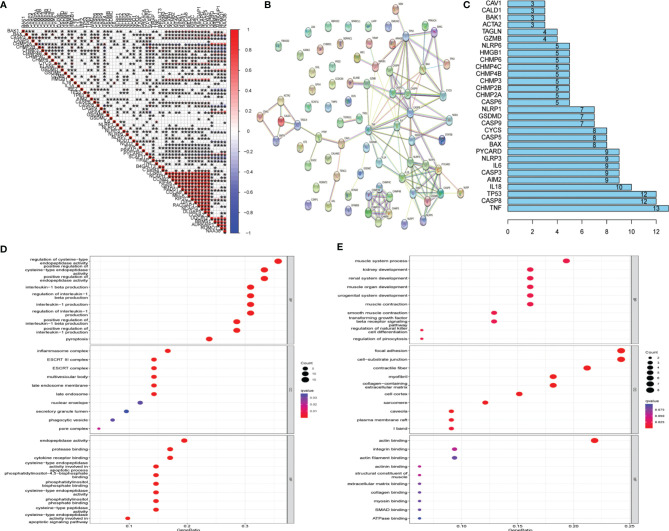
Protein–protein interaction (PPI) analysis and gene correlation analysis. **(A)** Correlation among the expression of 42 uterine corpus endometrial carcinoma (UCEC)-associated pyroptosis-related genes and 21 UCEC key genes. **(B)** The PPIs among 42 UCEC-associated pyroptosis-related genes and 21 UCEC key genes. **(C)** The rank of connection degree (number) for each gene. **(D)** Gene Ontology (GO) analysis for 42 UCEC-associated pyroptosis-related genes. **(E)** GO analysis for 21 UCEC key genes.

Twenty-one UCEC key genes and 42 UCEC-associated pyroptosis-related genes were examined. Gene correlation analysis for 21 UCEC key genes and 42 UCEC-associated pyroptosis-related genes was performed using TCGA data set (**Figure 3A**). The result showed that several pyroptosis-related genes have significant correlations with UCEC key genes. PPI analysis (**Figures 3B, C** **)** showed that these pyroptosis-related genes, especially *TNF, CASP8,* and *TP53*, could interact with these key UCEC genes.

To discover the potential molecular mechanisms of key UCEC genes (**Figure 3D**) and associated pyroptosis-related genes (**Figure 3E**), many involved biological processes were found using a GO enrichment analysis.

### Immune Landscape of Uterine Corpus Endometrial Carcinoma

**Figure 4:**
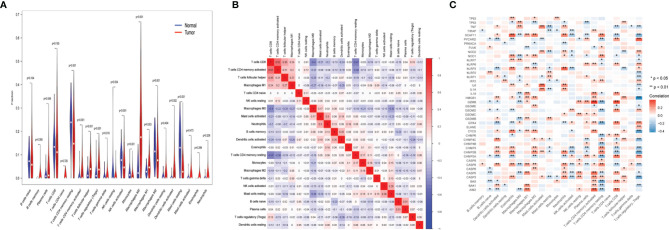
Immune landscape of uterine corpus endometrial carcinoma (UCEC). **(A)** The fraction of 22 types of immune cells in UCEC patients and normal cases. **(B)** The correlation of 22 types of immune cells in UCEC samples. **(C)** The correlation of 42 UCEC-associated pyroptosis-related gene expression and the abundance of immune cell infiltration. P-values are shown as: *p < 0.05; **p < 0.01.

The samples in TCGA data set were used for the CIBERSORT analysis. In **Figure 4A**, the Wilcoxon test used violin plots to analyze the proportion of 22 immune cell types in UCEC patients and normal cases using violin plots. As previously found, the distribution of resting memory CD4 T cells, activated memory CD4 T cells, follicular helper CD4 T cells, regulatory T cells (Tregs), gamma delta T cells, activated natural killer (NK) cells, monocytes, macrophages (M0, M1, and M2), activated dendritic cells, and resting mast cells showed significant variations in UCEC patients and normal cases. The results revealed that the UCEC and control groups have different immune environments. The next step was to assess the relevance of the 22 immune cells in the UCEC samples, and the result suggest that an interaction between the expression of immune cells occurred (**Figure 4B**). The correlation of 42 UCEC-associated pyroptosis-related gene expression and the abundance of immune cells was evaluated (**Figure 4C**). As previously found, the expression of 42 UCEC-associated pyroptosis-related genes may influence the infiltration of these immune cells.

### Exploration of the Expression of Pyroptosis-Related Genes in Each Tumor Grade

**Figure 5:**
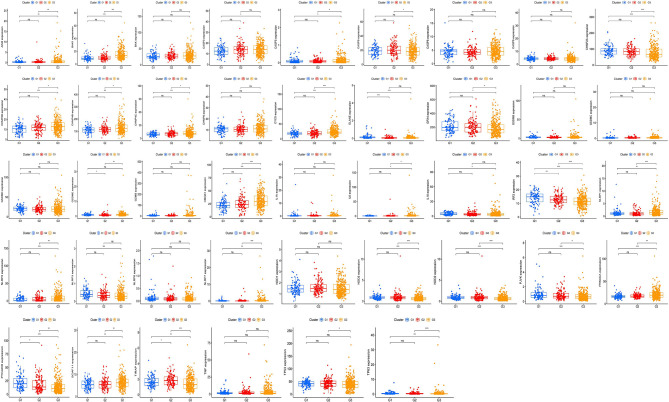
Exploration of the relationship between pyroptosis-related genes and tumor grade. The expression of 42 uterine corpus endometrial carcinoma (UCEC)-associated pyroptosis-related genes in each grade. P-values are shown as: *p <0.05; **p < 0.01; ***p < 0.001, ns means not statistically significant.

The expression of 42 UCEC-associated pyroptosis-related genes in each tumor grade (**Figure 5**) with the aim of assessing whether pyroptosis-related genes influence UCEC progression was examined. As the result showed, it was found that the expression of *PYCARD, TIRAP,* and *IRF2* had significant differences in different GOLD states, which suggested that these genes may significantly contribute to the progression of UCEC.

### Consensus Clustering of Pyroptosis-Related Genes Grouped Uterine Corpus Endometrial Carcinoma Into Two Clusters

**Figure 6:**
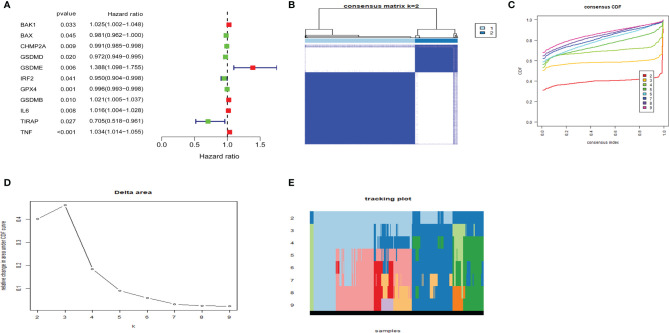
Consensus clustering of pyroptosis-related genes regrouped uterine corpus endometrial carcinoma (UCEC). **(A)** Identification of pyroptosis-related gene by univariate Cox regression analysis. **(B)** Correlation between Clusters 1 and 2. **(C)** Consensus clustering cumulative distribution function (CDF) for k = 2–9. **(D)** Relative change in area under CDF curve for k = 2–9. **(E)** The tracking plot for k = 2–9.

**Figure 7:**
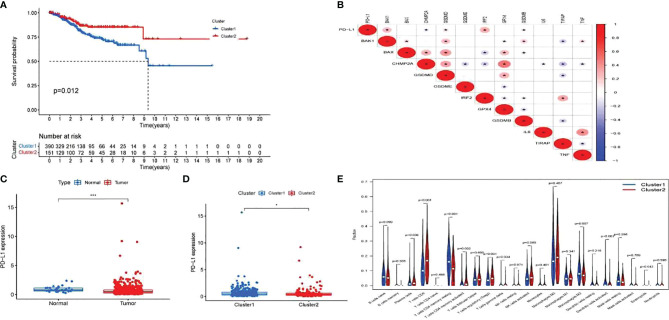
Exploration of the clinicopathological features and immunotherapeutic response on two clusters and correlation analysis. **(A)** Kaplan–Meier curve of overall survival (OS) difference between Clusters 1 and 2. **(B)** Correlation analysis of 12 pyroptosis-related gene and programmed cell death-ligand 1 (PD-L1). P-values are shown as: *p < 0.05. **(C)** PD-L1 expression between uterine corpus endometrial carcinoma (UCEC) patients and normal controls. P-values are shown as: *p < 0.05; ***p < 0.001. **(D)** PD-L1 expression between two clusters. **(E)** The fraction of 22 types of immune cells in Clusters 1 and 2.

Cox regression analysis was used to select pyroptosis-related prognostic genes. The result indicated that 11 pyroptosis-related genes apparently correlated with OS (**Figure 6A**). Using the similarities in the expression of pyroptosis-related genes, the value of k = 2 was selected (**Figures 6B–E**). UCEC samples were separated into two subgroups, namely, Clusters 1 and 2. As previously found, the OS of Cluster 1 subgroup was shorter than that of Cluster 2 (**Figure 7A**). The expression of immunomodulator programmed cell death-ligand 1 (*PD-L1*) was examined to study immunotherapeutic responses. Subsequently, correlation analysis validated the association among these genes. In addition, most pyroptosis-related genes correlated with *PD-L1* expression (**Figure 7B**). It was discovered that UCEC patients have a higher expression of *PD-L1* than that of normal people (**Figure 7C**), and patients in Cluster 1 have higher *PD-L1* expression than that of those in Cluster 2 (**Figure 7D**). The distribution of 22 immune cells in Cluster 1 and 2 groups is depicted in **Figure 7E**.

### Establishment and Verification of the Risk Model

**Figure 8:**
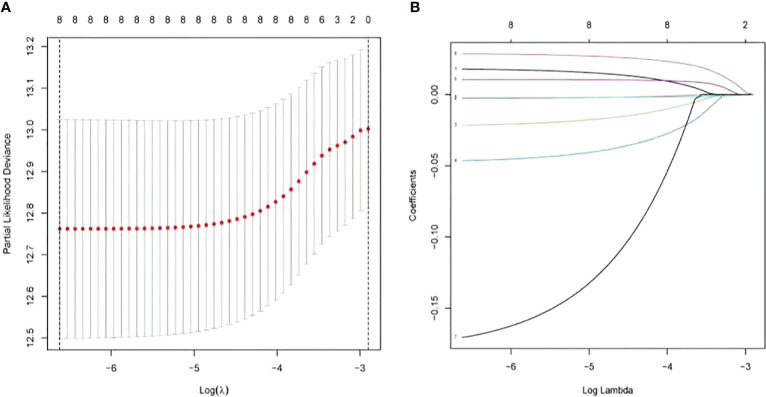
Construction of risk model based on pyroptosis-related genes by the least absolute shrinkage and selection operator (LASSO)-penalized Cox analysis.

**Figure 9:**
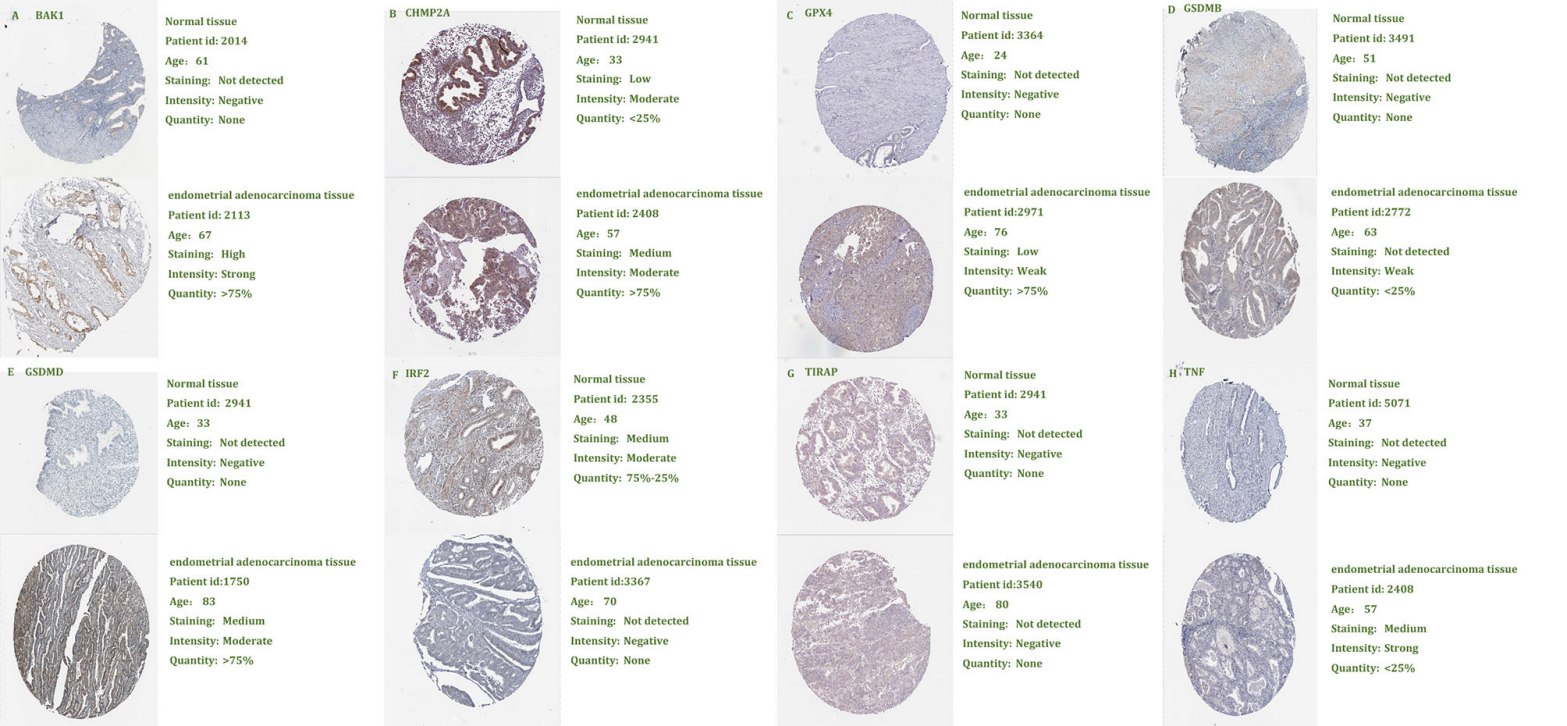
Validation of eight pyroptosis-related genes by The Human Protein Atlas database.

LASSO-penalized Cox analysis performs variable option and regularization concurrently. It is applied to choose optimal characters in high-dimensional data, which has inferior correlation and significant prediction value to avert overfitting. Furthermore, this method is extremely applicable for discerning the most available predictive marker and producing prognostic information relevant to clinical outcomes. The first rank value of log λ of the minimum segment likelihood deviation is illustrated by the broken vertical line. BAK1, CHMP2A, GSDMD, IRF2, GPX4, GSDMB, TIRAP, and TNF were selected to construct a prognosis risk model for UCEC (**Figure 8**). In addition, immunohistochemistry (IHC) data sets were retrieved from The Human Protein Atlas database that revealed the expression levels of pyroptosis-related proteins (**Figure 9**).

**Figure 10:**
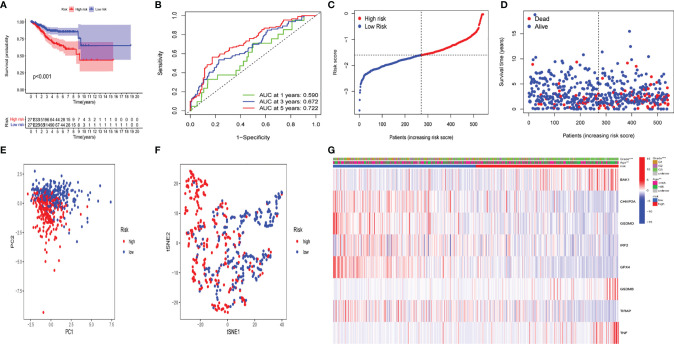
The prognostic value of the risk model in The Cancer Genome Atlas (TCGA) data set. **(A)** Kaplan–Meier survival curves of overall survival (OS) of patients in the high- and low-risk groups. **(B)** One-year, 3-year, and 5-year receiver operating characteristic (ROC) curves for OS prediction based on pyroptosis-related gene. **(C)** Risk grade distribution of risk model. **(D)** Different survival statuses and survival times between low- and high-risk groups. **(E)** Principal component analysis (PCA) of the overall RNA expression data between low-risk and high-risk groups. **(F)** T-distributed stochastic neighbor embedding (t-SNE) analysis of the overall RNA expression data between low- and high-risk groups. **(G)** Clinical evaluation by pyroptosis-related gene.

To demonstrate the prognostic power of the resulting model, a general formula was used to calculate the risk score for each patient. The risk score was calculated as follows: risk score = (BAK1 * 0.01789) + (CHMP2A* - 0.00270) + (GSDMD* - 0.02148) + (IRF2* - 0.04651) + (GPX4* - 0.00217) + (GSDMB *0.01053) + (TIRAP *-0.17035) + (TNF *0.02855). Based on the median value of risk scores, the samples were separated into two groups, including low- and high-risk groups. A Kaplan–Meier survival analysis indicated that the OS of UCEC patients with higher risk score was worse than that in lower risk score patients (**Figure 10A**). Subsequently, 1-, 3-, and 5-year receiver operating characteristic (ROC) curves were constructed to confirm the reliability of this risk model (**Figure 10B**). The distribution of risk scores, patterns of survival status, survival times, and principal component analysis and t-distributed stochastic neighbor embedding (PCA and t-SNE, respectively) of the overall RNA expression data between high- and low-risk groups was depicted (**Figures 10C–F**). Moreover, clinicopathological features of the two subgroups were compared (**Figure 10G**), and the results indicated that low- and high-risk groups have obvious distinctions in clinicopathological features, including age and tumor grades.

**Figure 11:**
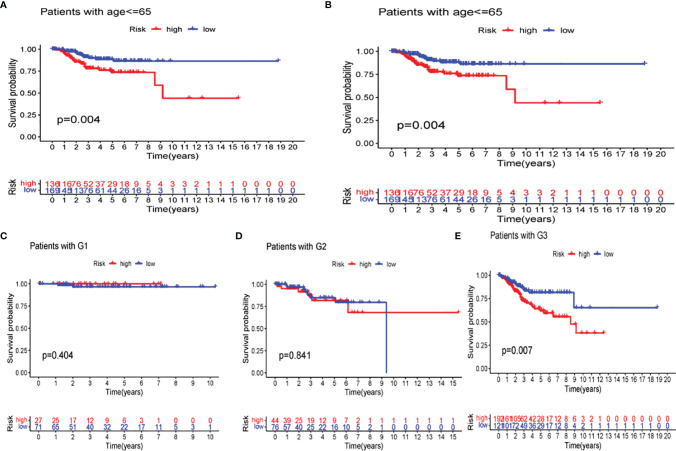
Kaplan–Meier curves of overall survival (OS) differences stratified by age **(A, B)** and tumor stage **(C–E)** between the low- and high-risk groups in The Cancer Genome Atlas (TCGA) data set.

The difference in several clinicopathological features with respect to OS between high- and low-risk groups was analyzed. As shown in **Figure 11**, based on the subgroups divided by age and tumor G3 grade, the OS of the low-risk group was superior to that of the high-risk group, but insignificant outcomes between tumor grades G1 and G2 were found.

### Evaluation of the Prognostic Risk Model

**Figure 12:**
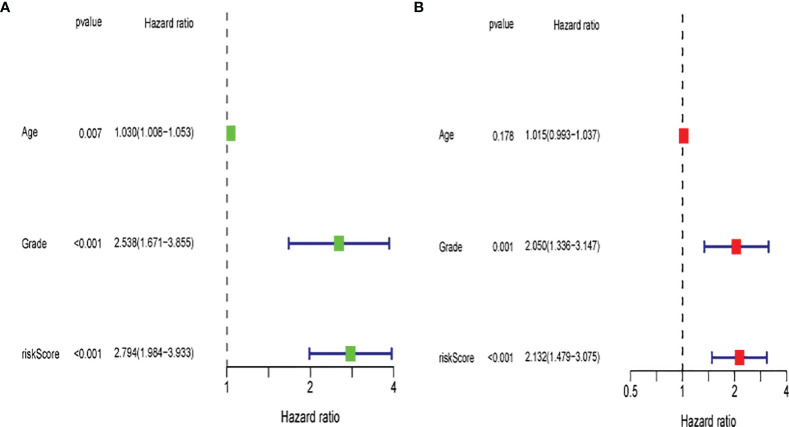
Evaluation of the prognostic risk model. (**A**) Univariate Cox regression analysis for the risk score. (**B**) Multivariate analysis for the risk score.

Univariate and multivariate Cox regression analyses were performed to assess the independence of the risk models based on the eight pyroptosis-related genes. For univariate Cox regression analysis, the hazard ratio (HR) and 95% confidence interval (CI) for risk score were 2.794 and 1.984–3.933 (P < 0.001) as shown in **Figure 12A**, and the results of multivariate Cox regression analysis showed an HR of 2.132 and 1.479–3.075 (P < 0.001) as shown in **Figure 12B**. It was concluded that the risk model of eight pyroptosis-related genes, independent of clinicopathological characteristics, including age and tumor grade, was the most accurate predictive factor for UCEC.

## Discussion

UCEC is the most frequently occurring gynecologic malignancy worldwide, and its mortality and morbidity rates are increasing (19). UCEC patients present similar clinical features, but they have different clinical outcomes due to molecular heterogeneity (20). Recently, several papers have reported that pyroptosis has a good diagnostic and predictive power as a biomarker in each type of UCEC (21, 22). In addition, pyroptosis has been reported to contribute a crucial effect to tumorigenesis and progression (23, 24). Therefore, a growing number of studies have focused on identifying the signature of pyroptosis to forecast survival and immunotherapeutic responses in UCEC.

Autophagy, pyroptosis, and other mechanisms have been proven to mediate the loss of cell viability (25). Pyroptosis plays a crucial role in a series of pathogenic processes (26, 27). The relationship between pyroptosis and a tumor is intricate with cell death both driving tumor progression and compromising antitumor immunity while inhibiting tumorigenesis (28). Yu et al. (29) found that γ-glutamyl hydrolase (GGH) expression levels were associated with Th2 cell expression and low-killer cell infiltration of CD56 bright cells, a process that could drive UCEC progression. Zheng et al. (30) found that *NPAS2* could lead to UCEC *via* an increase in tumor immune cell infiltration. Liu et al. (31) developed and validated a Treg-related risk signature (TRRS) to assess the prognosis of UCEC and reflect the immune status of UCEC; this *TRRS* was capable of predicting the prognosis of UCEC patients that allowed for personalized treatment to be provided. After a rigorous review of the references, it was concluded that pyroptosis was related to the development and progression of inflammatory or malignant tumors.

In recent advances in medicine, immune checkpoint inhibitors have proven to be a key therapeutic measure for malignancies (32). Programmed death protein 1 (*PD-1*), PD-L1, PD-L2, and cytotoxic T lymphocyte-associated protein 4 (*CTLA4*) are common immune checkpoints (33, 34). *PD-L1* is extensively expressed throughout the body, especially in cancer cells and immune cells (3). In our prognostic model, quantification of immune checkpoints in different clusters revealed higher expression in UCEC patients than that in normal subjects and higher expression in cluster 1 patients than that in cluster 2, suggesting that UCEC patients and cluster 1 patients could probably benefit from immunotherapy. Subsequently, the samples were divided into high- and low-risk groups based on the median of the risk scores. Further exploring the potential correlation between pyroptosis-related genes and immune infiltration, the analysis suggested that the high-risk group was characterized by lower levels of immune cell infiltration (monocytes, resting mast cells). Furthermore, as our study found, the immune scores of the high-risk group were lower than those of the low-risk group, suggesting a potential difference in their immune environment.

UCEC patients always have poor survival clinical results, emphasizing the requirement for more credible biomarkers of long-term patient prognosis and therapy (35). In our study, 47 pyroptosis-related genes were identified to explore the prognostic function of pyroptosis-related genes. Forty-two pyroptosis-related genes were confirmed for their function in UCEC development and progression. Eight pyroptosis-related genes were used to establish pyroptosis-related gene models to predict the OS of UCEC patients. Among them, *IRF2* was shown to trigger the activation of GSDMD by mediating pyroptosis (36). Inflammatory vesicle activation initiates focal death *via* recruitment of caspase-1. *GSDMD*, a caspase-1/11 substrate, contributed to the promotion of the formation of non-selective pores within the plasma membrane, thereby inducing pyroptosis, which in turn leads to cell swelling, rupture, and release of pro-inflammatory factors, for instance, *HMGB1, ATP,* and *IL-1β* (12, 37, 38). Recently, pyroptosis was found to be activated by different molecular mechanisms, in which *GSDMB* is the executioner, but not *GSDMD* (39). Meanwhile, *GSDMD* and *GSDMB* were differentially expressed in most tumors, and all evidence supported their involvement in the induction of the pyroptosis process in cancer, which also increased the predictive value of UCEC (40, 41). *GPX4* was a classical selenoprotein with lipid peroxidation inhibitory properties, which belonged to the glutathione peroxidase family (42). *GPX4* played a role in the induction of cell death in a variety of cancers (43–45). Guerriero et al. (46) showed that GPX4 inhibits macrophage pyroptosis in mice. For UCEC, it may be beneficial to increase GPX4 and thus inhibit pyroptosis. In this study, *GPX4* expression levels were found to be reduced in the high-risk group. Given the role of *GPX4* in pyroptosis, the development of inducers targeting *GPX4* may reduce the incidence of UCEC associated with pyroptosis and thus improve patient survival. It has been previously reported that *CHMP2A* deficiency leads to the accumulation of autophagic vesicles and induces pyroptosis (47). However, its role in cancer has not been clearly studied to date. Our study showed that low expression of CHMP2A was associated with poor prognosis in UCEC patients, which suggested that inhibition of *CHMP2A* deletion could be a target for the treatment of UCEC. In addition, other pyroptosis-related genes were mentioned for the first time in UCEC.

The differences in OS between the high-risk and low-risk groups according to age and tumor G3 grading were analyzed, with better OS in the low-risk than that in the high-risk group, but no significant results in tumor G1 and G2 grading were found. ROC analysis revealed that this model outperformed a model based on conventional clinical characteristics for predicting survival in UCEC. The risk model based on eight pyroptosis-related genes associated with OS was quite accurate. In this study, the prognostic model that can validly predict the prognosis of endometrial cancer in patients was constructed using the genes related to pyroptosis as a starting point. In our study, we used several methods to determine this new model to ensure its optimality and rational use. These results provide insight into future studies of the processes and mechanisms by which pyroptosis-related genes affect UCEC onset, development, and prediction. Currently, limited progress in the study of pyroptosis has been made, and the relationship between UCEC and pyroptosis has not been investigated. Although this particular relationship was explored to some extent and a prognostic model from multiple perspectives was constructed and validated, some shortcomings and limitations in our study should be recognized. First, this study used retrospective data, which may have some heterogeneity among patients. Therefore, more prospective cohort studies in larger populations are needed to test the prognostic value of this risk model. Second, more extensive molecular experiments should be performed to demonstrate the function of pyroptosis-related genes. Therefore, clinical sample size will be expanded to attempt to demonstrate the accuracy of the prediction model through further external validation to probe the interaction between pyroptosis-related genes and UCEC.

In conclusion, our study investigated UCEC occurrence, development, and prognosis and may contribute to reveal the course and mechanism of pyroptosis correction.

## Data Availability Statement

The original contributions presented in the study are included in the article/**Supplementary Material**. Further inquiries can be directed to the corresponding authors.

## Author Contributions

XH, YL, and FX have contributed to the design of the study, searching the related papers and extracting data, preparing figures, analysis and interpretation of the data, and drafting the article. JL, XY, and JX took part in searching the related papers and extracting data, preparing figures, and analysis and interpretation of data. All authors read and approved the final submitted article.

## Funding

This study was supported by grants from the Guangdong Basic and Applied Basic Research Foundation (2020A1515011519), Science and Technology Special Fund Project of Guangdong Province (High-level Hospital Construction Project) (2021010303), (Shanfu [2021] No. 88), the “Dengfeng Project” for the construction of high-level hospitals in Guangdong Province–The First Affiliated Hospital of Shantou University Medical College Supporting Funding (2019-70), and the Medical Science and Technology Research Foundation of Guangdong Province (A2020430, A2021409).

## Conflict of Interest

The authors declare that the research was conducted in the absence of any commercial or financial relationships that could be construed as a potential conflict of interest.

## Publisher’s Note

All claims expressed in this article are solely those of the authors and do not necessarily represent those of their affiliated organizations, or those of the publisher, the editors and the reviewers. Any product that may be evaluated in this article, or claim that may be made by its manufacturer, is not guaranteed or endorsed by the publisher.

## Supplementary Material

Supplementary Figure 1 Flowchart of this study.
