# Supplementary figures and images for: The Expression of Pyroptosis-Related Gene May Influence the Occurrence, Development, and Prognosis of Uterine Corpus Endometrial Carcinoma
Source: Front Oncol. 2022 Apr 29;12:885114. doi: 10.3389/fonc.2022.885114 (PMC9103195; doi:10.3389/fonc.2022.885114)

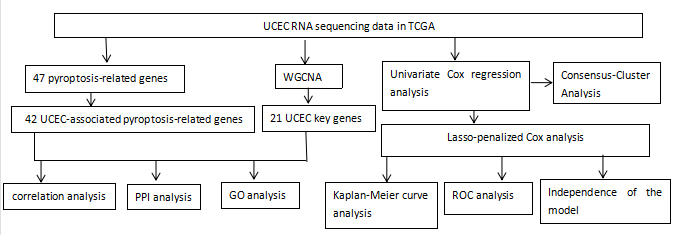

Supplement: Supplementary Figure 1 — Flowchart of this study. [file Image_1.tif]
